# Supplementary material for: A prediction model of working memory across health and psychiatric disease using whole-brain functional connectivity
Source: eLife. 2018 Dec 10;7:e38844. doi: 10.7554/eLife.38844 (PMC6324880; doi:10.7554/eLife.38844)
Supplement: Supplementary file 1. [file elife-38844-supp1.docx]

**Supplementary file 1. Resting state fMRI scan parameters and their values.**

| **Parameter** | **SCZ** | | **MDD** | | | **OCD** | | **ASD** | | | |  |
| --- | --- | --- | --- | --- | --- | --- | --- | --- | --- | --- | --- | --- |
| Site | KYU, scanner 1 | KYU, scanner 2 | | HRU,  scanner 1 | HRU,  scanner 2 | | KPU | | UTK | UTK | SHU | |
| Machine | Siemens Tim Trio | Siemens Trio | | GE  Signa HDxt | Siemens Magnetom | | Philips Achieva | | Philips Achieva | Philips Achieva | GE  Signa | |
| Number of patients/controls | 5/16 | 53/44 | | 31/30 | 46/33 | | 46/47 | | 0/33 | 33/0 | 36/38 | |
| Magnetic field strength (T) | 3 | 3 | | 3 | 3 | | 3 | | 3 | 3 | 1.5 | |
| Field of view (mm) | 212 | 256 | | 256 | 192 | | 192 | | 224 | 220 | 220 | |
| Matrix | 64 × 64 | 64 × 48 | | 64 × 64 | 64 × 64 | | 64 × 64 | | 64 × 64 | 80 × 80 | 64 × 64 | |
| Number of slices | 40 | 30 | | 32 | 38 | | 39 | | 45 | 34 | 27 | |
| Number of volumes | 240 | 177 | | 150 | 112 | | 200 | | 200 | 200 | 204 | |
| In-plane resolution (mm) | 3.3125 ×  3.3125 | 4.0 ×  4.0 | | 4.0 ×  4.0 | 3.0 ×  3.0 | | 3.0 ×  3.0 | | 3.5 × 3.5 | 2.75 × 2.75 | 3.4375 × 3.4375 | |
| Slice thickness (mm) | 3.2 | 4 | | 4 | 3 | | 3 | | 3.5 | 5 | 5 | |
| Slice gap (mm) | 0.8 | 0 | | 0 | 0 | | 0 | | 0 | 0 | 1 | |
| TR (ms) | 2,500 | 2,000 | | 2,000 | 2,700 | | 2,000 | | 2,500 | 2,500 | 2,000 | |
| TE (ms) | 30 | 30 | | 27 | 31 | | 30 | | 30 | 30 | 30 | |
| Total scan time (mm:ss) | 10:00 | 6:00 | | 5:00 | 5:03 | | 6:40 | | 8:20 | 8:20 | 6:48 | |
| Flip angle (deg) | 80 | 90 | | 90 | 90 | | 80 | | 75 | 75 | 90 | |

SCZ data is shared except for part of data KYU scanner 2 patients (*N* = 3) and control (*N* = 1).

MDD data is shared except for part of data: HRU scanner 2 controls (*N* = 5), HRU scanner 1 patients (*N* = 9), HRU scanner 2 patients (*N* = 16).

OCD data is shared except for patients (*N* = 43) and controls (*N* = 47).

ASD data is not shared.
