# Supplementary material for: A prediction model of working memory across health and psychiatric disease using whole-brain functional connectivity
Source: eLife. 2018 Dec 10;7:e38844. doi: 10.7554/eLife.38844 (PMC6324880; doi:10.7554/eLife.38844)
Supplement: Supplementary file 2. [file elife-38844-supp2.docx]

**Supplementary file 2. Whole-brain intrinsic functional network labels and their component regions.**

| **Rank** | **Network** | **Cluster** | **Brain Regions** |
| --- | --- | --- | --- |
| 1 | Left fronto-parietal network | Fronto-parietal | left-lateralized fronto-parietal regions |
| 2 | Middle frontal and parietal network | Motor/visuospatial | middle frontal gyri and superior parietal lobules |
| 3 | Supplemental motor network | Motor/visuospatial | superior and middle frontal gyri including Supplemental and premotor areas as well as frontal eye field |
| 4 | Right fronto-parietal network | Fronto-parietal | right-lateralized fronto-parietal regions |
| 5 | Cingulo-opercular network | Emotion/interoception | bilateral anterior insula/frontal opercula and the anterior aspect of the body of the cingulate gyrus |
| 6 | Lateral temporal network | Visual | middle and inferior temporal gyri |
| 7 | Lateral occipital network | Visual | lateral posterior occipital cortices |
| 8 | Basal ganglia | Emotion/interoception | bilateral basal ganglia and thalamus |
| 9 | Primary sensorimotor network (mouth) | Audition/speech | dorsal precentral gyri, central sulci, postcentral gyri, superior and inferior cerebellum |
| 10 | Midbrain | Emotion/interoception | midbrain |
| 11 | Primary sensorimotor network (hand) | Motor/visuospatial | ventral precentral gyri, central sulci, postcentral gyri, superior and inferior cerebellum |
| 12 | Cerebellum | Cerebellum | cerebellum |
| 13 | Medial occipital network | Visual | medial posterior occipital cortices |
| 14 | Orbitofrontal network | Emotion/interoception | subgenual anterior cingulate cortex and orbitofrontal cortex |
| 15 | Auditory | Audition/speech | transverse temporal gyri |
| 16 | Default mode network | Default-mode | medial prefrontal and posterior cingulate/precuneus areas |
| 17 | Limbic | Emotion/interoception | limbic and medial-temporal areas |
| 18 | Superior parietal network | Motor/visuospatial | superior parietal lobule |

Rank indicates relevance with working memory function according to the BrainMap ICA.
